# Serum uric acid distribution according to *SLC22A12 *W258X genotype in a cross-sectional study of a general Japanese population

**DOI:** 10.1186/1471-2350-12-33

**Published:** 2011-03-02

**Authors:** Nobuyuki Hamajima, Mariko Naito, Asahi Hishida, Rieko Okada, Yatami Asai, Kenji Wakai

**Affiliations:** 1 Department of Preventive Medicine, Nagoya University Graduate School of Medicine, Nagoya, Japan; 2 Seirei Social Welfare Community, Hamamatsu, Japan

## Abstract

**Background:**

Although *SLC22A12 258X *allele was found among those with hypouricemia, it was unknown that serum uric acid distribution among those with *SLC22A12 258X *allele. This study examined serum uric acid (SUA) distribution according to *SLC22A12 *W258X genotype in a general Japanese population.

**Methods:**

Subjects were 5,023 health checkup examinees (3,413 males and 1,610 females) aged 35 to 69 years with creatinine < 2.0 mg/dL, who were participants of a cohort study belonging to the Japan Multi-Institutional Collaborative Cohort Study (J-MICC Study). *SLC22A12 *W258X was genotyped with a polymerase chain reaction with confronting two-pair primers.

**Results:**

The genotype frequency was 4,793 for *WW*, 225 for *WX*, and 5 for *XX*, which was in Hardy-Weinberg equilibrium (p = 0.164) with *X *allele 0.023 (95% confidence interval [0.021-0.027]). Mean (range) SUA was 6.2 (2.1-11.4) mg/dL for *WW*, 3.9 (0.8-7.8) mg/dL for *WX*, and 0.8 (0.7-0.9) mg/dL for *XX *among males, and 4.5 (1.9-8.9) mg/dL, 3.3 (2.0-6.5) mg/dL, and 0.60 (0.5-0.7) mg/dL among females, respectively. Six individuals with SUA less than 1.0 mg/dL included two males with *XX *genotype, one male with *WX *genotype, and three females with *XX *genotype. Subjects with *WX *genotype were 14 (77.8%) of 18 males with a SUA of 1.0-2.9 mg/dL, and 28 (34.6%) of 81 females with the same range of SUA. The corresponding values were 131 (25.1%) of 522 males and 37 (3.5%) of 1,073 females for SUA 3.0-4.9 mg/dL, and 8 (0.4%) of 2,069 males and 5 (1.1%) of 429 females for SUA 5.0-6.9 mg/dL. The *X *allele effect for SUA less than 3 mg/dL was significantly (p < 0.001) higher in males (OR = 102.5, [33.9-309.8]) than in females (OR = 25.6 [14.4-45.3]).

**Conclusions:**

Although *SLC22A12 *W258X was a determining genetic factor on SUA, SUA of those with *WX *genotype distributed widely from 0.8 mg/dL to 7.8 mg/dL. It indicated that other genetic traits and/or lifestyle affected SUA of those with *WX *genotype, as well as those with *WW *genotype.

## Background

It is well known that the mean serum uric acid (SUA) is lower in females than in males. In addition, it is fully documented that age, menopause, food consumption, alcohol intake, obesity, a sedentary lifestyle, dyslipidemia, insulin resistance, blood pressure, renal function, and drug use for hypertension were associated with SUA levels [1-6]. Meanwhile, recent studies have elucidated that genotypes are also influential factors of SUA.

SUA is reabsorbed in renal tubules through uric acid transporter 1 (URAT1) encoded by *SLC22A12 *in chromosome 11q13 [7,8]. *SLC22A12 *was documented to have functional polymorphisms, among which W258X was found among those with renal hypouricemia [9-11]. However, the distribution of SUA among those with *258X *allele was not reported in a general population. This study aimed to examine SUA distribution according to *SLC22A12 *W258X genotye, elucidating the overall effect of *SLC22A12 *W258X on SUA among Japanese.

## Methods

This study was approved by the Ethics Committee of Nagoya University School of Medicine (approval number 288). Subjects who gave written informed consent to participate in the study were enrolled.

### Subjects and data collection

Subjects were derived from 5,040 examinees aged 35-69 years who visited a health checkup center in Hamamatsu, Japan in 2006-2007. They were enrolled as participants of a cohort study belonging to the Japan Multi-Institutional Collaborative Cohort Study (J-MICC Study) [12,13]. As of October, 2010, one participant was found to be ineligible in terms of age (34 years old at enrollment), and 11 participants withdrew from the study. Blood sample was not available for one participant, and genotyping was not successful for another. Three participants with creatinine of 2.0 mg/dL or over were excluded from the analysis, leaving 5,023 subjects for the analysis.

Health checkup data including blood tests were used for this study. Peripheral blood was drawn in the morning from those fasting overnight. Biochemical analysis of the sampled sera was performed using an auto-analyzer in the health checkup center.

### Genotyping

**Figure 1:**
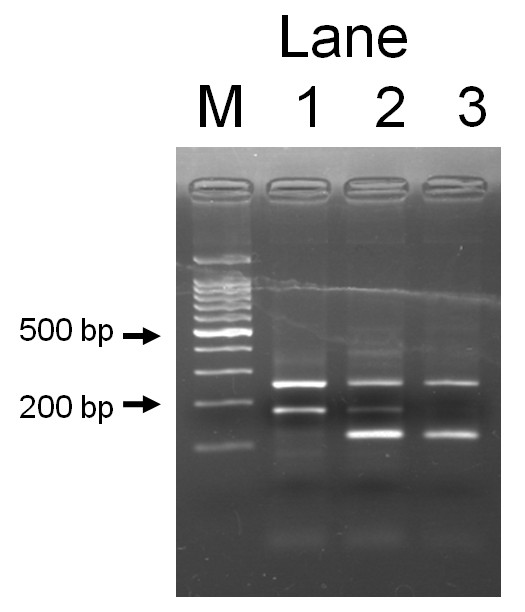
**Representative gel for *SLC22A12 *W258X polymorphism**. Lane M, a 100 bp ladder; lane 1, a *WW *homozygote with fragments of 117 bp and 255 bp; lane 2, a *WX *heterozygote with fragments of 117 bp and 176 bp and 255 bp; lane 3, a *XX *homozygote with fragments of 176 bp and 255 bp.

DNA was extracted from buffy coat conserved at -80°C using a BioRobot^® ^M48 (QIAGEN Group, Tokyo). *SLC22A12 *W258X polymorphism was genotyped by a polymerase chain reaction with confronting two-pair primers (PCR-CTPP) [14]. Each 25 μl reaction tube contained 30-80 ng DNA, 0.12 mM dNTP, 12.5 pmol of each primer, 0.5 U AmpliTaq Gold (Perkin-Elmer, Foster City, CA) and 2.5 μl of 10x PCR buffer including 15 mM MgCl_2_. The PCR-CTPP was conducted with initial denaturation at 95°C for 10 minutes, 35 cycles of denaturation at 95°C for 1 minute, annealing at 62°C for 1 minute, and extension at 72°C for 1 minute, and a final extension at 72°C for 5 minutes. The primers were F1: 5'- TCC ATG CAG GCT CCA GG -3', R1: 5'- ACC ACC AGC TGC AGC AGT GTT -3', F2: 5'- TAC GGT GTG CGG GAC TGG -3', and R2: 5'- GGC AGG ATC TCC TCT GAG G -3'. The amplified DNA fragments were 117-base pairs (bp) for the *W *allele (*G *allele), 176-bp for the *X *allele (*A *allele), and 255-bp for a common band, as demonstrated in Figure 1.

### Statistical analysis

Body mass index (BMI) was calculated by weight (kg)/squared height (m^2^). Hardy-Weinberg equilibrium was examined with a chi-square test. Binomial distribution was used to estimate 95% confidence interval (CI) of proportions. Means among the three genotype groups were tested with analysis of variance (ANOVA). Adjusted odds ratio (OR) and 95% CI were estimated using an unconditional logistic model. All statistical analyses were performed using STATA software version 11 (STATA, College Station, TX).

## Results

Table 1 shows the characteristics of the 5,023 subjects (3,413 males and 1,610 females). Those with a SUA less than 3.0 mg/dL were 0.6% in males and 5.2% in females, while those with a SUA of 7.0 mg/dL or over were 23.5% in males and 1.5% in females. The genotype frequency was 4,793 for *WW*, 225 for *WX*, and 5 for *XX*, which was in Hardy-Weinberg equilibrium (p = 0.164); the *X *allele frequency was 0.023 (95% CI, 0.021-0.027).

**Table 1:** Characteristics of subjects according to sex

Characteristics	Males		Females	
		
	N	(%)	N	(%)
Total	3,413	(100)	1,610	(100)
Age (years)				
35-39	265	(7.8)	192	(11.9)
40-49	983	(28.8)	495	(30.7)
50-59	1,366	(40.0)	630	(39.1)
60-69	799	(23.4)	293	(18.2)
SUA (mg/dL)				
-3.0	21	(0.6)	84	(5.2)
3.0-4.9	522	(15.3)	1,073	(66.6)
5.0-6.9	2,069	(60.6)	429	(26.6)
7.0-	801	(23.5)	24	(1.5)
BMI (kg/m^2^)*				
-18.4	90	(2.6)	160	(9.9)
18.5-24.9	2,423	(71.0)	1,195	(74.2)
25.0-	899	(26.3)	255	(15.8)
Creatinine (mg/dL)				
0.0-0.4	0	(0.0)	33	(2.1)
0.5-0.9	2,710	(79.4)	1,574	(97.8)
1.0-1.4	701	(20.5)	3	(0.2)
1.5-1.9	2	(0.1)	0	(0.0)
*SLC22A12 *W258X				
*WW*	3,256	(95.4)	1,537	(95.5)
*WX*	155	(4.5)	70	(4.3)
*XX*	2	(0.1)	3	(0.2)

SUA, serum uric acid; BMI, body mass index

* BMI was not available for one male.

The means of age, BMI, blood pressure, and blood tests according to the genotype and sex are listed in Table 2. The null hypothesis that the means were equal among the three genotypes was rejected with one-way ANOVA for creatinine and glucose in males, and for age and gamma-glutamyltransferase (GGT) in females. When the *WX *and *XX *were combined, the difference in the mean between the combined and the *WW *became nonsignificant for GGT in females, but still significant for creatinine and glucose in males and age in females.

**Table 2:** Characteristics of subjects according to *SLC22A12 *W258X genotype

Characteristics	Males				Females			
		
	*WW*	*WX*	*XX*	P	*WW*	*WX*	*XX*	p
	n = 3,256	n = 155	n = 2		n = 1,537	n = 70	n = 3	
Age (years)	50.6	52.0	52.5	0.166	49.1	51.4	58.3	0.017
Body mass index (kg/m^2^)	23.5	23.7	20.9	0.240	22.0	21.9	19.7	0.407
Systolic blood pressure (mmHg)	121.0	120.5	105.0	0.319	114.4	115.1	119.3	0.811
Diastolic blood pressure (mmHg)	76.3	76.0	66.0	0.350	69.9	70.4	66.7	0.798
Total cholesterol (mg/dL)	202.3	202.7	195.5	0.944	207.2	208.9	197.3	0.793
HDL cholesterol (mg/dL)	57.7	56.0	63.5	0.338	71.1	69.7	88.3	0.159
Triglyceride (mg/dL)	124.7	129.0	66.0	0.460	85.1	85.2	73.3	0.895
AST (U/dL)	22.4	22.4	18.0	0.890	19.5	20.5	27.0	0.071
ALT (U/L)	25.5	26.6	18.5	0.732	17.1	19.5	24.7	0.066
GGT (U/L)	47.0	45.9	24.5	0.817	23.2	25.0	56.3	0.023
Creatinine (mg/dL)	0.86	0.82	0.90	0.001*	0.61	0.62	0.63	0.750
Blood urea nitrogen (mg/dL)	14.3	14.5	16.0	0.567	13.1	14.0	15.0	0.057
Blood glucose (mg/dL)	101.9	107.0	90.5	0.003*	93.7	95.2	89.7	0.434

HDL, high-density lipoprotein; AST, aspartate aminotransferase; ALT, alanine aminotransferase; GGT, gamma-glutamyltransferase

p-value was calculated from one-way analysis of variance among *WW*, *WX*, and *XX *genotypes.

**Figure 2:**
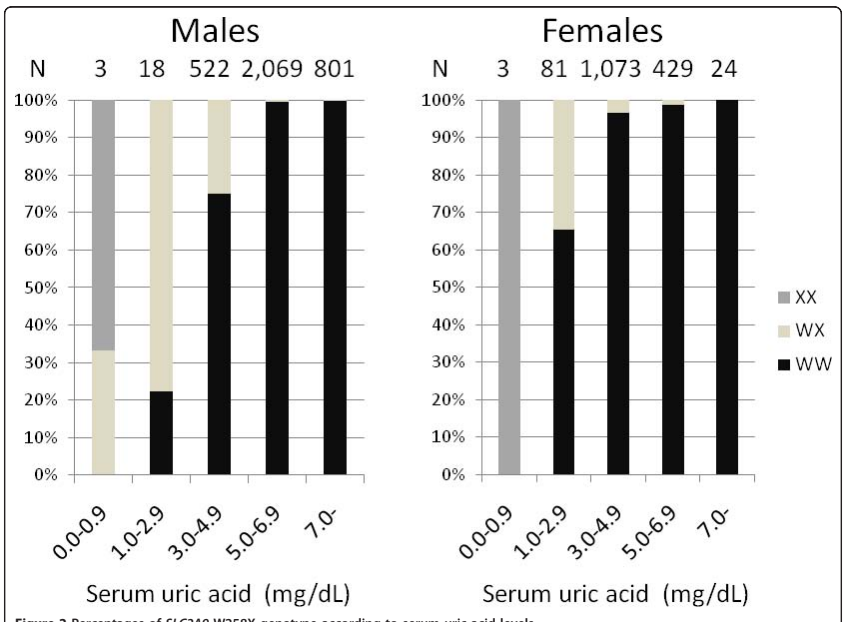
**Percentages of *SLC2A9 *W258X genotype according to serum uric acid levels**.

Mean SUA was 6.21 mg/dL for *WW*, 3.95 mg/dL for *WX*, and 0.80 mg/dL for *XX *among males, and 4.50 mg/dL, 3.31 mg/dL, and 0.60 mg/dL among females, respectively. The difference in mean SUA between *WX *and *WW *genotypes was significantly (p = 8E-12) higher in males (2.26 mg/dL) than in females (1.19 mg/dL). The difference among three genotypes was highly significant both in males (p < 1E-40) and females (p = 1E-33). Table 3 demonstates the SUA distribution in percentage according to the genotype. All five individuals with *XX *genotype had a SUA of less than 1.0 mg/dL; the range was 0.7 mg/dL to 0.9 mg/dL in males and 0.5 mg/dL to 0.7 mg/dL in females. The SUA of those with *WX *genotype varied from 0.8 mg/dL to 7.8 mg/dL in males and from 2.0 mg/dL to 6.5 mg/dL in females, while the corresponding values for those with *WW *genotype were 2.1 mg/dL to 11.4 mg/dL in males and 1.9 mg/dL to 8.9 mg/dL in females. Subjects with *WX *genotype were 14 (77.8%) of 18 males with SUA 1.0-2.9 mg/dL, and 28 (34.6%) of 81 females with the same range of SUA. The corresponding values were 131 (25.1%) of 522 males and 37 (3.5%) of 1,073 females for SUA 3.0-4.9 mg/dL, and 8 (0.4%) of 2,069 males and 5 (1.1%) of 429 females for SUA 5.0-6.9 mg/dL, as depicted in Figure 2.

**Table 3:** Serum uric acid (SUA) distribution (%) according to *SLC22A12 *W258X among Japanese health checkup examinees

Genotype	N	SUA (mg/dL)											
		
		0.0-0.9	1.0-1.9	2.0-2.9	3.0-3.9	4.0-4.9	5.0-5.9	6.0-6.9	7.0-7.9	8.0-8.9	9.0-	Mean	**S.D**.
Males													
*WW*	3,256	0.0	0.0	0.1	1.6	10.4	31.4	31.9	17.4	6.1	1.0	6.21	1,12
*WX*	155	0.6	0.0	9.0	40.0	44.5	3.2	1.9	0.6	0.0	0.0	3.95	0.83
*XX*	2	100.0	0.0	0.0	0.0	0.0	0.0	0.0	0.0	0.0	0.0	0.80	0.14
Total	3,413	0.1	0.0	0.5	3.3	12.0	30.1	30.5	16.7	5.9	0.9	6.10	1.21
													
Females													
*WW*	1,537	0.0	0.1	3.4	24.9	42.5	22.8	4.7	1.2	0.3	0.0	4.50	0.94
*WX*	70	0.0	0.0	40.0	38.6	14.3	5.6	1.4	0.0	0.0	0.0	3.31	0.89
*XX*	3	100.0	0.0	0.0	0.0	0.0	0.0	0.0	0.0	0.0	0.0	0.60	0.10
Total	1,610	0.2	0.1	5.0	25.5	41.2	22.0	4.6	1.2	0.3	0.0	4.44	0.98

* 95% CI: 95% confidence interval for SUA mean

The age-adjusted OR (95% CI) of SUA < 3 mg/dL for the *X *allele was significantly (p < 0.001) higher in males (OR = 102.5, 95% CI, 33.9-309.8) than in females (OR = 25.6, 95% CI, 14.4-45.3). The corresponding ORs were reduced for SUA < 4 mg/dL, as shown in Table 4. Among those with BMI < 25 kg/m^2^, the age-adjusted OR (95% CI) was 116.3 (32.5-416.8) in 2,514 males and 23.3 (12.7-42.9) in 1,355 females, while they were 78.6 (8.4-731.5) in 899 males with BMI > 25 kg/m^2 ^and 56.2 (9.9-320.4) in 255 females with BMI > 25 kg/m^2^. When these ORs were adjusted for creatinine and glucose in males and for GGT in females, no substantial differences were observed; for example, the OR of SUA < 3 mg/dL for *X *allele was 89.8 instead of 102.5 in males and 30.7 instead of 25.6 in females.

**Table 4:** Odds ratio (OR) and 95% confidence interval (95% CI) of low serum uric acid (SUA) for *SLC22A12 *W258X among Japanese health checkup examinees

	SUA < 3 mg/dL				SUA < 4 mg/dL			
		
Genotype	< 3 mg/dL	3 mg/dL ≤	OR	95% CI	< 4 mg/dL	4 mg/dL ≤	OR	95% CI
Males								
*WW*	4	3,252	1	Reference	55	3,201	1	Reference
*WX *or *XX*	17	140	102.5	33.9-309.8	79	78	58.2	38.6-87.9
Total	21	3,392			157	3,279		
								
Females								
*WW*	53	1,484	1	Reference	436	1,101	1	Reference
*WX *or *XX*	31	42	25.6	14.4-45.3	58	15	11.9	6.6-21.5
Total	84	1,526			494	1,116		
								
Both sexes								
Males with *WW*	4	3,252	1	Reference	55	3,201	1	Reference
Males with *WX *or *XX*	17	140	106.5	35.3-321.6	79	78	63.7	42.0-96.5
Females with *WW*	53	1,484	27.2	9.8-75.4	436	1,101	22.4	16.8-29.9
Females with *WX *or *XX*	31	42	672.6	225.4-2006.8	58	15	246.3	130.9-363.7
Interaction*			0.23	0.07-0.80			0.17	0.08-0.35
Total	105	4,918			628	4,395		

* Interaction between females and *X *allele for low SUA.

## Discussion

This study demonstrated the SUA distribution according to *SLC22A12 *W258X genotype in a general Japanese population. The SUA of all five subjects with *XX *genotype was less than 1 mg/dL, while SUA of those with *WX *genotype distributed widely; from 0.8 mg/dL to 7.8 mg/dL in males and from 2.0 mg/dL to 6.5 mg/dL in females. The difference in the mean SUA between *WX *and *WW *genotypes was 2.26 mg/dL in males and 1.19 mg/dL in females, indicating that the reduction of the mean SUA due to possessing *X *allele was significantly larger in males than in females. Since the distribution among the males with *WX *genotype was closer to that among the females with *WW *genotype (Table 3), the effect of *X *allele on SUA was similar to the effect of sex difference. BMI did not significantly modify the effect of the *X *allele on SUA both for males and females. These findings were actually new for the associations between *SLC22A12 *W258X and SUA.

Among Japanese, the frequency of *258X *allele was 0.024 among 1,875 participants of a cohort study [11], 0.023 among 980 controls in a case-control study [15], and 0.025 among 5,165 participants from another cohort study [16], which were quite similar to the estimate in the present study (0.023). Among Koreans, *258X *allele was found in 3 of 5 hypouricemia patients [17], and was 1.1% in a general population [18]. Since this allele has not been reported among other ethnic groups to date, the origin was thought to be in East Asia [19].

Several genotypes affecting SUA have been reported to date. *ABCG2 *in chromosome 4q22 coding ATP-binding cassette subfamily G member 2 has functional polymorphisms, Q126X (rs72552713) and Q141K (rs2231142), with a minor allele frequency of 0.018 and 0.281 in Japanese, respectively [20]. Although the genotypes with reduced function increase the risk of hyperuricemia [20-22], *126X *was rare and *141K *was less influential on SUA. *SLC2A9 *in chromosome 4p16-p15.3 coding glucose transporter 9 (GLUT9) was reported to have mutations (R380W and R198C in Japanese [23], L75R in an Israeli-Arab family, and exon 7 deletion in Ashkenazi-Jewish [24]) causing hypouricemia. Their allele frequency was very rare. Common polymorphisms including *MTHFR *C677T have been reported to have an association with SUA [25,26], but the impact was limited in comparison with the above genotypes. Accordingly, *SLC22A12 258X *seemed to be one of the important genetic traits influencing SUA among Japanese.

The present study discovered that the *X *allele had a significantly larger impact in males than in females. The OR of possessing the *X *allele was larger in males than in females; 102.5 vs 25.6 for SUA < 3 mg/dL and 58.2 vs 11.9 for SUA < 4 mg/dL. The differences in mean SUA between males and females were 0.20 mg/dL among those with *XX *genotype, 0.63 mg/dL among those with *WX *genotype, and 1.71 mg/dL among those with *WW *genotype. There was no biological explanation for these phenomena.

In the present study, since the subjects with *XX *genotype were few, the distriubtion of those with *XX *genotype might not reflect the distribution of the population with *XX *genotype. Another limitation was that the medication influencing SUA was not taken into account for the genotype frequency according to the SUA level. Since the medication was common for hyperuricemia, but not for hypouricemia, the effect due to the medication might be limited for low SUA.

## Conclusions

In conclusion, this study demonstrated the SUA distribution according to *SLC22A12 *W258X genotype in a large study. The effect of *X *allele was larger in males than in females. Since the *X *allele was influential and relatively common among Japanese, the information on the genotype would be useful for the interpretation of individual SUA. Since SUA distributes widely among Japanese with *WX *genotype, further studies are warranted to elucidate the determinants of the SUA distribution among those with *WX *genotype, as well as among those with *WW *genotype.

## List of abbreviations

BMI: body mass index; bp: base pairs; CI: confidence interval; GLUT9: glucose transporter 9; OR: odds ratio; PCR-CTPP: polymerase chain reaction with confronting two-pair primers; SUA: serum uric acid; URAT1: uric acid transporter 1.

## Competing interests

The authors declare that they have no competing interests.

## Authors' contributions

NH conceived of the study, participated in the design and coordination, and drafted the manuscript. MN and KW participated in the design and coordination, edited the data, and drafted the manuscript. RO contributed the genotyping, establishing PCR primers and PCR conditions for *SLC22A12 *W258X. AH and YA participated in the coordination, organizing the informed consent process and data/sample collection. All authors read and approved the final manuscript.

## Pre-publication history

The pre-publication history for this paper can be accessed here:

http://www.biomedcentral.com/1471-2350/12/33/prepub
